# The CANWARD studies: Canadian Antimicrobial Resistance Alliance (CARA) Canadian Hospital Ward Antibiotic Resistance Surveillance collection

**DOI:** 10.1093/jac/dkaf265

**Published:** 2025-08-28

**Authors:** James C Hurley, Jean-Yves Madec

**Affiliations:** Melbourne Medical School, University of Melbourne, Parkville, Victoria, Australia; Ballarat Clinical School, Deakin University, Ballarat, Victoria, Australia; Internal Medicine Service, Ballarat Health, Grampians Health Services, Ballarat, Victoria, Australia; Paris, France

This is the third supplement reporting on the Canadian ward surveillance study (CANWARD).^1,2^ CANWARD provides surveillance for commonly tested and recently approved antimicrobials, for bacterial pathogens obtained from more than 15 tertiary care Canadian hospitals representing at least 8 of the 10 Canadian provinces.

This supplement contains nine articles, starting with an introductory commentary by Zhanel and Adam, and provide an overview of the 17 years of CANWARD and some of the notable findings.

The current reports emanating from this surveillance appear in this supplement. It updates surveillance information published in *Journal of Antimicrobial Chemotherapy* in 2019.^2^

It details targeted surveillance information for Gram-positive pathogens, Gram-negative pathogens and other specific pathogen categories for isolates that were obtained from inpatients and outpatients attending medical wards, emergency rooms, ICUs, hospital clinics and surgical wards in Canadian hospitals. A small proportion were isolates from paediatric patients. The reports detail the inclusion criteria but the aim was to obtain susceptibility data broadly representative of pathogens seen across the breadth of Canada over the surveillance period. The surveillance provides antimicrobial resistance trends since 2007. There is also information of trends in relation to serotype patterns in *Streptococcus pneumoniae*.

The editors hope that this information will serve as a benchmark for antimicrobial resistance in Canada and a reference to serve as a model for similar surveillance studies elsewhere in the world.
